# Metabonomics applied in exploring the antitumour mechanism of physapubenolide on hepatocellular carcinoma cells by targeting glycolysis through the Akt-p53 pathway

**DOI:** 10.1038/srep29926

**Published:** 2016-07-15

**Authors:** Ting Ma, Bo-Yi Fan, Chao Zhang, Hui-Jun Zhao, Chao Han, Cai-Yun Gao, Jian-Guang Luo, Ling-Yi Kong

**Affiliations:** 1 State Key Laboratory of Natural Medicines, Department of Natural Medicinal Chemistry, China Pharmaceutical University, 24 Tong Jia Xiang, Nanjing 210009, China

## Abstract

Metabolomics can be used to identify potential markers and discover new targets for future therapeutic interventions. Here, we developed a novel application of the metabonomics method based on gas chromatography-mass spectrometry (GC/MS) analysis and principal component analysis (PCA) for rapidly exploring the anticancer mechanism of physapubenolide (PB), a cytotoxic withanolide isolated from *Physalis* species. PB inhibited the proliferation of hepatocellular carcinoma cells *in vitro* and *in vivo*, accompanied by apoptosis-related biochemical events, including the cleavage of caspase-3/7/9 and PARP. Metabolic profiling analysis revealed that PB disturbed the metabolic pattern and significantly decreased lactate production. This suggests that the suppression of glycolysis plays an important role in the anti-tumour effects induced by PB, which is further supported by the decreased expression of glycolysis-related genes and proteins. Furthermore, the increased level of p53 and decreased expression of p-Akt were observed, and the attenuated glycolysis and enhanced apoptosis were reversed in the presence of Akt cDNA or p53 siRNA. These results confirm that PB exhibits anti-cancer activities through the Akt-p53 pathway. Our study not only reports for the first time the anti-tumour mechanism of PB, but also suggests that PB is a promising therapeutic agent for use in cancer treatments and that metabolomic approaches provide a new strategy to effectively explore the molecular mechanisms of promising anticancer compounds.

Cancer cells have unique metabolic characteristics related to altered glucose metabolism known as the Warburg effect in which aerobic glycolysis is increased^1^. In normal cells, glycolysis is prioritized only when the oxygen supply is limited. However, cancer cells preferentially ferment glucose into lactate to generate energy even in the presence of sufficient oxygen^2^,^3^. The switch towards glycolysis provides a selective growth advantage that supports their rapid proliferation and expansion across the body^4^. Therefore, interfering with aerobic glycolysis represents a potentially effective strategy to selectively target cancer cells^5^. As previously reported, the activation of the PI3K/Akt pathway and the inactivation of the tumour suppressor p53 protein have both been suggested to contribute to the Warburg effect^6^,^7^,^8^. Akt mediates the negative control of p53 levels through enhancing the mouse double minute 2 (MDM2)-mediated targeting of p53 for degradation^9^. These findings suggest that understanding the role of the Akt-p53 pathway is important in the study of glycolysis in tumour cells.

Metabolomics involves the unbiased quantitative and qualitative analysis of the full complement of low molecular weight metabolites present in cells, body fluids and tissues based on modern analytical techniques with high throughput, sensitivity and resolution, such as mass spectrometry and nuclear magnetic resonance (NMR) spectroscopy^10^. Over the past decades, metabolomics has been used to identify metabolic alterations in various types of diseases and as a way to indicate early biological changes in the host due to perturbations in metabolic pathways. The emerging field of metabolome analysis promises immense potential for biomarker discovery and the exploration of molecular mechanisms in perturbed systems related to diseases, providing a rapid way to look for targeted molecular markers and pathways^11^.

Withanolides have attracted much scientific attention due to their structural features and important pharmacological activities such as antimicrobial, anticancer, anti-inflammatory, and immunomodulatory effects^12^,^13^,^14^. Physapubenolide (PB, Fig. 1A) is a withanolide that was first isolated from *Physalis pubescens*^15^. In our previous study, PB was found to possess potent cytotoxic activity and exhibited potential as a promising therapeutic agent for use in the treatment of cancer^16^. However, the anticancer mechanism of PB is not fully understood. In the present study, we report for the first time on the anti-tumour mechanism of PB and the application of metabolomics during the process of exploring the molecular mechanism of the antitumour activity of PB. Our results demonstrate that metabolomics is an effective method for exploring the complex mechanisms of natural products and that PB induces apoptosis and a decrease in the level of glycolysis through the Akt-p53 pathway.

## Results

### PB decreased the proliferation of hepatocellular carcinoma cells

**Figure 1:**
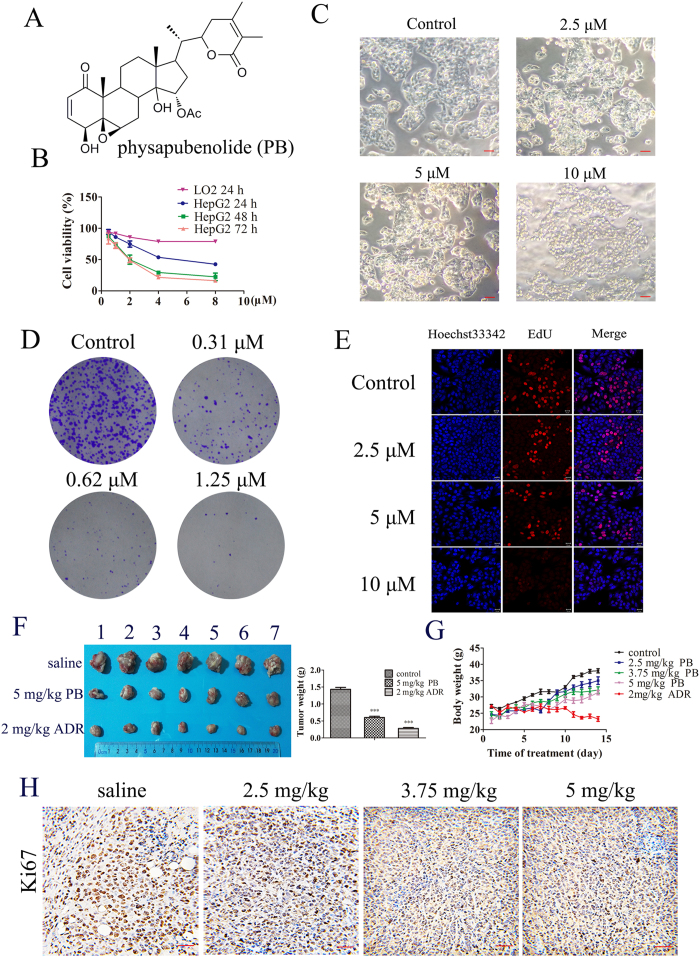
PB decreased the proliferation of hepatocellular carcinoma cells and arrested tumour growth *in vivo*. (**A**) The chemical structure of physapubenolide. (**B**) HepG2 cells were treated with different concentrations of PB, and then the cell viability was measured via an MTT assay at indicated time point. (**C**) The morphological changes of HepG2 cells after treatment with PB for 24 h. Bar, 50 μm. (**D**) The effect of PB on the clonogenic ability in the HepG2 cells. (**E**) HepG2 cells were incubated with various concentrations of PB for 24 h, EdU staining was then performed and the cells were observed using confocal microscopy. (**F**) The size and weight changes of tumour when the mice were administered 5 mg/kg PB or 2 mg/kg Adr. (**G**) The body weight changes in the mice treated with PB and Adr. (**H**) Immunohistochemical staining for Ki-67 in tumour tissues obtained from the control and PB-treated mice. Bar, 20 μm.

To estimate the anti-proliferative effect of PB on hepatocellular carcinoma (HCC) cells^16^, HepG2 cells were chosen. Exposure to increasing concentrations (0.5–8 μM) of PB resulted in dose- and time-dependent decreases in HepG2 cell viability at different time points (24 h, 48 h, 72 h) with an IC_50_ of 5.31 ± 0.23, 2.52 ± 0.07 and 2.33 ± 0.08 μM, respectively. However, when the LO2 cells were treated with PB (0.5–8 μM), the inhibition rates were lower than 20%, suggesting a selective proliferation inhibition of PB on cancer cells (Fig. 1B). As for cell morphology, HepG2 cells in the control group grew well, while the cells treated with the various concentrations of PB had indistinct shapes or were even round and showed an increase in floating cells (Fig. 1C), suggesting an obvious decrease in cell viability. The effect of PB on the colony forming ability of HepG2 cells was also investigated, and a significant reduction in clonogenic ability at 0.31 μM and 0.62 μM PB and an almost complete cessation of colony formation at 1.25 μM was observed (Fig. 1D). To further confirm the effect of PB on cell proliferation, an EdU incorporation assay was conducted, and we observed that PB significantly reduced the number of EdU-positive cells compared with number in the DMSO treated group in a dose-dependent manner (Fig. 1E). Taken together, these data indicate that PB indeed inhibits the proliferation of human hepatocellular carcinoma cells.

### PB arrested tumour growth *in vivo*

To further verify the cancer cell proliferation inhibiting effect of PB, a mouse-xenograft model bearing liver carcinoma H22 cells was established to evaluate the antitumour effect of PB *in vivo*. Adriamycin (Adr) was used as a positive control. The data demonstrate that PB significantly inhibited tumour growth, as indicated by reduced tumour weight, and that it had little toxic effect on the mice. However, the weight of mice that were administrated Adr was obviously reduced compared to the weight of the control group, indicating the serious toxicity of Adr in mice (Fig. 1F,G). A histological analysis of the organs was performed following H&E staining. The control and PB treated groups showed no obvious toxic reaction, but the Adr-treated group exhibited severe toxicity of the heart and spleen (Fig. S1). In addition, the Ki-67 assay also demonstrated that PB inhibited HCC cell proliferation (Fig. 1H). Collectively, these results indicate PB has potential tumour-inhibiting effects and minimal toxic effects on the mice.

### PB distinctly affected glycolysis pathway both *in vitro* and *in vivo*

**Figure 2:**
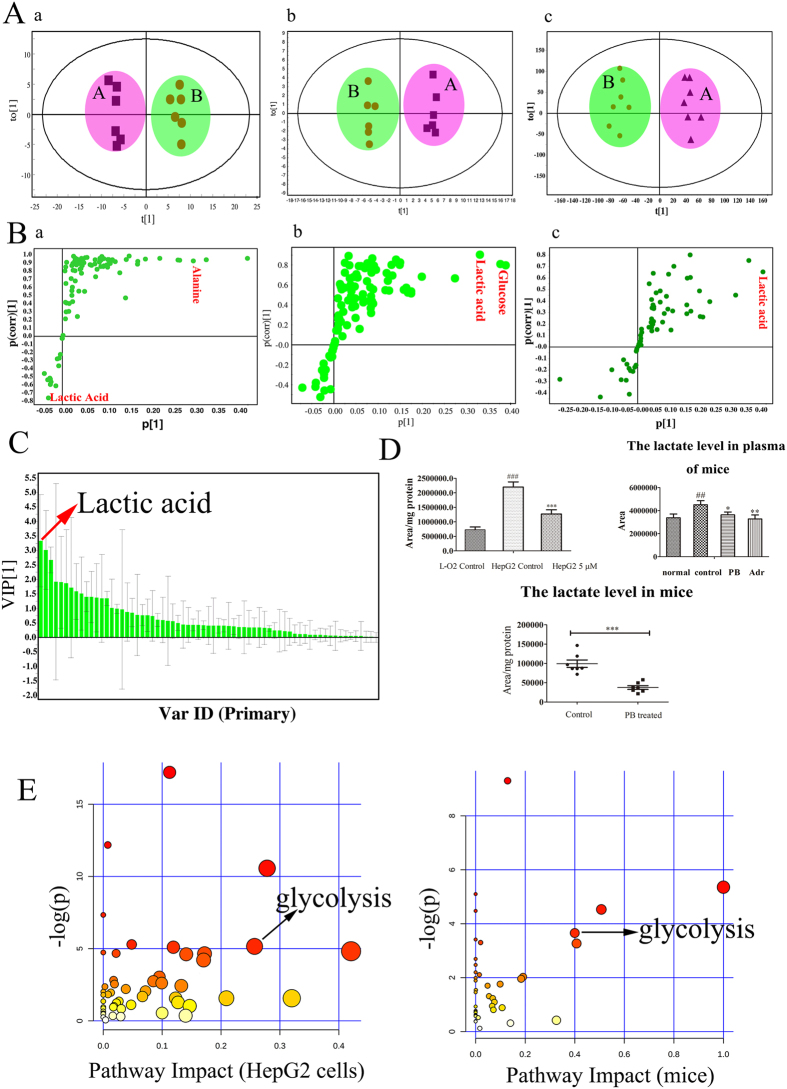
PB distinctly affected glycolysis pathway both *in vitro* and *in vivo*. (**A**) The OPLS-DA plot based on the intracellular metabolites in PB-treated or untreated HepG2 cells and in the tumour tissues and plasma of mice. a: HepG2 cells; b: tumour tissues of mice; c: plasma of mice. (A-control, B-PB treated) (**B**) S-plot in response to the constructed OPLS-DA model of HepG2 cells and mice. a: HepG2 cells; b: tumour tissues of mice; c: plasma of mice. (**C**) VIP score plot in response to the constructed OPLS-DA model of HepG2 cells. Targeted metabolites with a VIP score larger than 1 were considered to be potential biomarkers in discriminating control and PB-treated samples. (**D**) The lactate changes in HepG2 cells and the plasma and tissues of mice determined by GC-MS. (**E**) Metabolic pathway analyses of HepG2 cells and mice based on the variable metabolites of significant difference in HepG2 cells and tumour tissues.

To evaluate the effect of PB on the HepG2 cells and mice and to explore the targeted pathways, a metabolomics analysis of animal and cell samples was performed. The GC/MS total ion chromatogram (TIC) of the plasma and tumour tissues samples obtained from mice are shown in Fig. S2. A PCA plot based on the metabolites of mice and cells shows that the different groups of mice and cells clustered closely within each group and separately from each other, indicating a different metabolic pattern among them. Exposure to PB markedly perturbed the HepG2 metabolic pattern but had little effect on the pattern of the LO2 cells, suggesting that the effect of PB on LO2 cells might be weaker than on HepG2 cells (Figs 2A and S3A). The heat map also reflected the differences in metabolic substances between HepG2 and LO2 cells and that PB disturbed the metabolism of HepG2 cells (Fig. S4). An orthogonal partial least-squares discriminant analysis (OPLS-DA) was carried out to look for potential metabolic markers. Among the metabolites, lactic acid, glucose and alanine were obviously changed, with high values of | p [1] | or | p (corr) [1] | (Fig. 2B). Lactate showed the greatest variation of the important metabolites identified from the OPLS-DA plot, illustrating that alterations to glycolysis play an important role in the anti-cancer effects of PB (Fig. 2C). Statistical analyses and metabolite identification also revealed that metabolite levels related with glycolysis were severely perturbed when HepG2 cells were exposed to PB. For example, HepG2 cells had a higher level of lactate than the LO2 cells, and PB decreased it *in vivo* and *in vitro* (Fig. 2D). The amount of malic acid, glucose, and citric acid also decreased, demonstrating that HepG2 cells suffered from improper glucose metabolism. Additionally, the levels of amino acids all increased after the HepG2 cells were treated with 5 μM PB (Fig. S3B). The metabolic pathway analysis of the most variable metabolites further demonstrated that PB disturbed glycolysis pathway (Fig. 2E). Taken together, the results of the metabolomic profiling analysis indicate that PB significantly perturbs the glycolysis pathway both *in vitro* and *in vivo*.

### PB induced a decreased level of glycolysis both *in vitro* and *in vivo*

**Figure 3:**
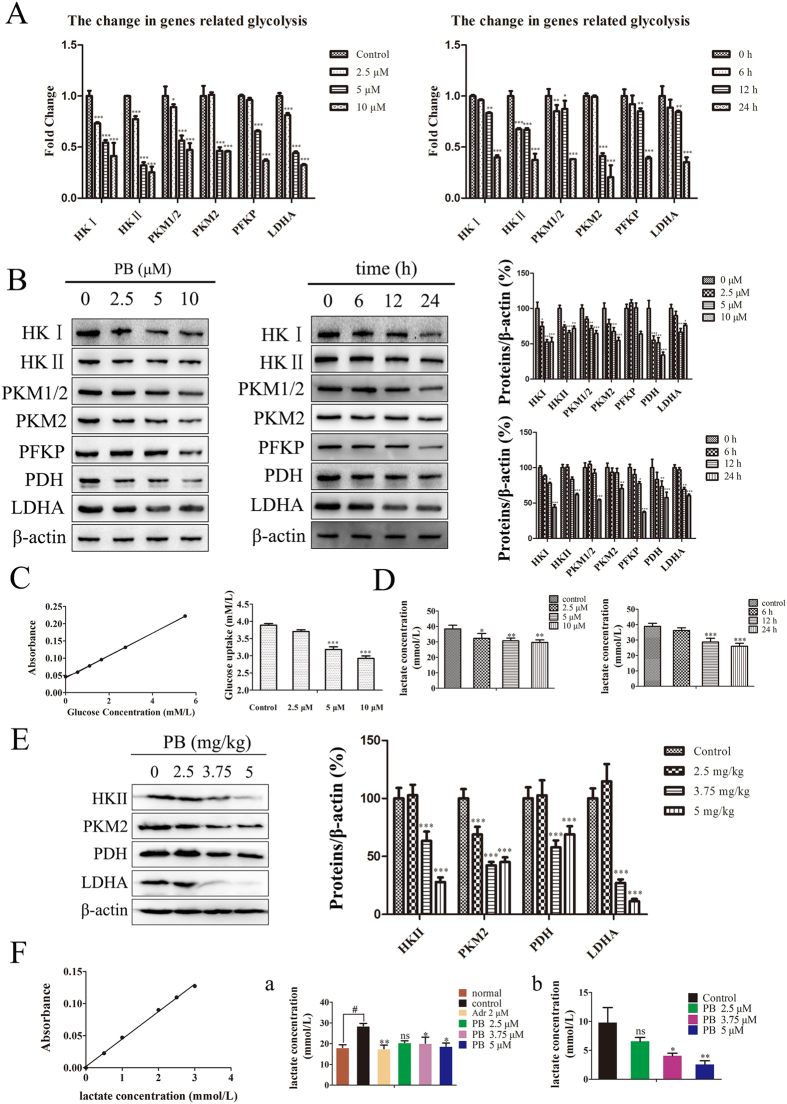
PB induced a decreased level of glycolysis both *in vitro* and *in vivo*. (**A**) The gene expression level of HKI, HKII, PKM1, PKM2, PFKP and LDHA in HepG2 cells was measured via quantitative real-time RT-PCR. (**B**) The protein expressions of HKI, HKII, PKM1, PKM2, PFKP and LDHA in HepG2 cells after treatment with various concentrations of PB for 24 h and 5 μM PB for different time intervals, as determined by western blotting. (**C**) The glucose in the medium was quantitated using a glucose assay kit to assess the glucose uptake of HepG2 cells treated with PB. (**D**) The lactate in the medium was measured via a lactate assay kit when HepG2 cells were treated with different concentrations of PB and 5 μM PB for different time intervals. (**E**) The protein levels of HKII, PKM2, PDH and LDHA in mice treated with or without PB, as determined by western blotting. (**F**) The lactate level in the plasma (a) and tissues (b) of mice treated with different concentrations of PB or Adr.

Because PB significantly disturbed the glycolytic pathway and reduced lactate production, as shown in the metabolomics analysis, the role of PB on glycolysis was further examined. The mRNA level of hexokinase I (HKI), hexokinase II (HKII), pyruvate kinase isoform M1 (PKM1), pyruvate kinase isoform M2 (PKM2), phosphofructokinase (PFKP), and lactate dehydrogenase-A (LDHA) all decreased when the HepG2 cells were incubated with 2.5 μM, 5 μM and 10 μM PB for 24 h or 5 μM PB for different time intervals (Fig. 3A). In addition to the gene levels, we also evaluated protein expression when HepG2 cells were cultured in medium in the absence or presence of PB. As shown in Fig. 3B, we found that levels of proteins related to glycolysis were significantly reduced compared with their levels in the control group in a time- and concentration-dependent manner. Furthermore, glucose uptake decreased in HepG2 cells following exposure to different concentrations of PB (Fig. 3C). Lactic acid is a compound representative of the glycolytic process, and it also decreased in both a dose- and time-dependent manner in HepG2 cells, as measured by a lactate kit (Fig. 3D). In mice, the expression of HKII, PKM2, pyruvate dehydrogenase (PDH) and LDHA all decreased in PB-treated groups (Fig. 3E), and the lactate levels were reduced in the plasma and tumour tissues of PB-treated mice relative to the levels in the saline-treated group (Fig. 3F). Therefore, these data demonstrate that PB decreases the glycolytic level in tumour cells.

### PB triggered mitochondrial apoptosis *in vitro* and *in vivo*

**Figure 4:**
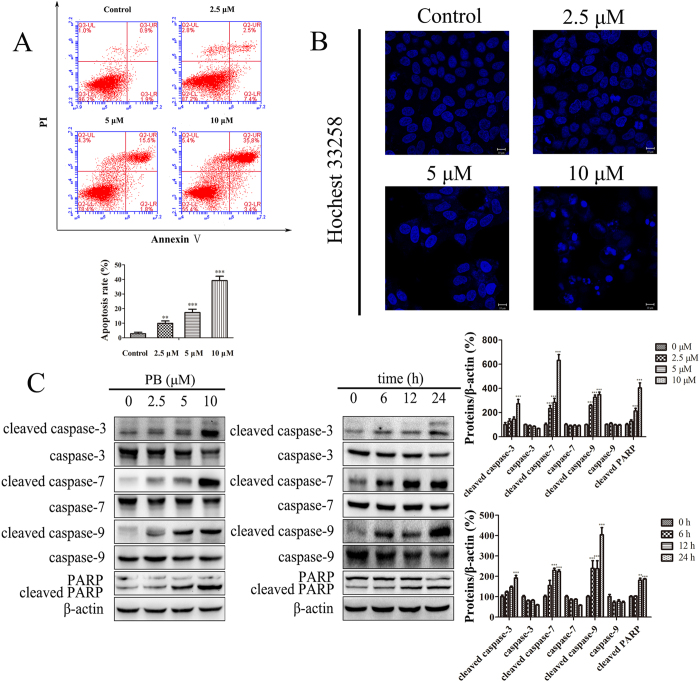
PB triggered mitochondrial apoptosis *in vitro* and *in vivo*. (**A**) The apoptotic rate of HepG2 cells after treatment with PB for 24 h, as determined by AnnexinV- FITC and PI staining. (**B**) HepG2 cells were stained with Hoechst 33258 after treatment with different concentrations of PB for 24 h and observed using confocal microscopy. (**C**) The expression of cleaved caspase-3, caspase-3, cleaved caspase-7, caspase-7, cleaved caspase-9, caspase-9, and cleaved PARP in HepG2 cells after treatment with various concentrations of PB for 24 h and 5 μM PB for different time intervals.

**Figure 5:**
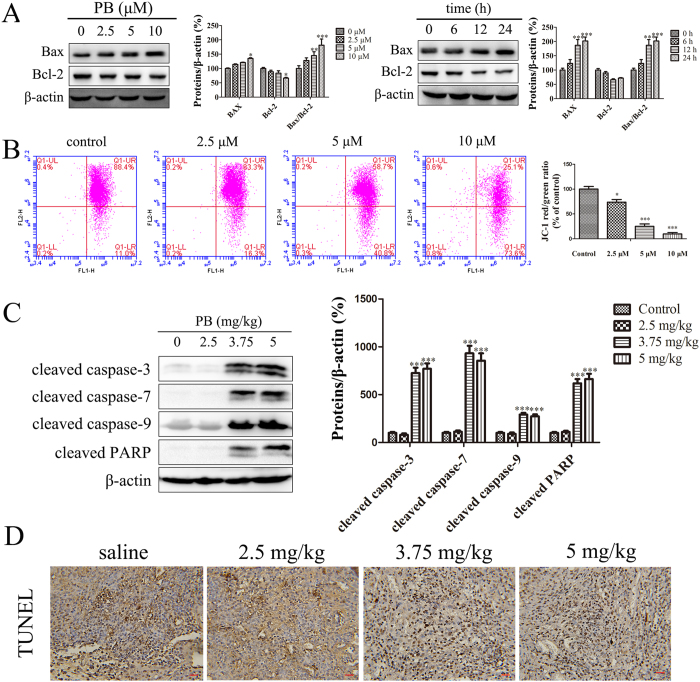
PB triggered mitochondrial apoptosis *in vitro* and *in vivo*. (**A**) The levels of Bax and Bcl-2 when HepG2 cells were incubated with 0, 2.5, 5 and 10 μM PB and 5 μM PB for 0, 6, 12 and 24 h. (**B**) The mitochondrial membrane potential of HepG2 cells treated with PB for 24 h, as measured via flow cytometry with JC-1 staining. (**C**) The protein levels of cleaved caspase-3, cleaved caspase-7, cleaved caspase-9 and cleaved PARP in mice treated with PB. (**D**) Apoptosis of tumour cells in mice treated with different doses of PB, as determined by TUNEL assay. Bar, 20 μm.

It has been reported that inhibiting glycolysis can induce cancer cells apoptosis^17^, and the glycolytic inhibitor 2-DG could be a potential choice in the treatment of cancer^1^. In our experiment, an increased protein expression of cleaved caspase-3/7/9 and PARP was detected when the HepG2 cells were treated with 10, 20 and 30 mM 2-DG (Fig. S4). Because of the inhibitory effects of PB on glycolysis, the changes in apoptotic markers were analysed when HepG2 cells were treated with PB. First, after PB treatment, HepG2 cells were labelled with AnnexinV- FITC antibodies and propidium iodide (PI). The results of this labelling show that 10% of the cells were apoptotic when treated with 2.5 μM PB for 24 h, and as the concentrations reached 10 μM, the amount of apoptotic cells increased to 39.2% (Fig. 4A), indicating the occurrence of PB-induced apoptosis. Apoptotic cells show characteristic nuclear shrinkage and fragmentation. Therefore, apoptosis was further confirmed by staining HepG2 cells with Hoechst 33258. As shown in Fig. 4B, more of the PB-treated cells exhibited obvious chromatin condensation and chromatinorrhexis when compared with the control cells. Finally, the levels of the caspase protein family were measured. In our experiments, the levels of cleavage-activated caspase-3, caspase-7, caspase-9 and PARP were observed to be significantly increased in both a dose- and time- dependent manner (Fig. 4C). In addition to the induced expression of caspase proteases, an alteration in the Bcl-2/Bax protein ratio is also involved in mitochondrial apoptosis. In our experiment, an increased Bax/Bcl-2 protein ratio was also observed after cells were treated with PB (Fig. 5A). Moreover, PB dramatically decreased the mitochondrial membrane potential, suggesting that mitochondrial dysfunction was involved in the observed apoptosis (Fig. 5B). The expression of cleaved caspase-3/7/9 and PARP was also enhanced in mice treated with PB, suggesting the occurrence of apoptosis *in vivo* (Fig. 5C). The results of the transferase-mediated deoxyuridine triphosphate-biotin nick-end labelling (TUNEL) assay also revealed that apoptosis was induced by PB in mice (Fig. 5D). In conclusion, these data suggest that PB induces mitochondrial apoptosis *in vitro* and *in vivo*.

### The effect of PB on metabolic pattern disruption was regulated through the Akt-P53 pathway

**Figure 6:**
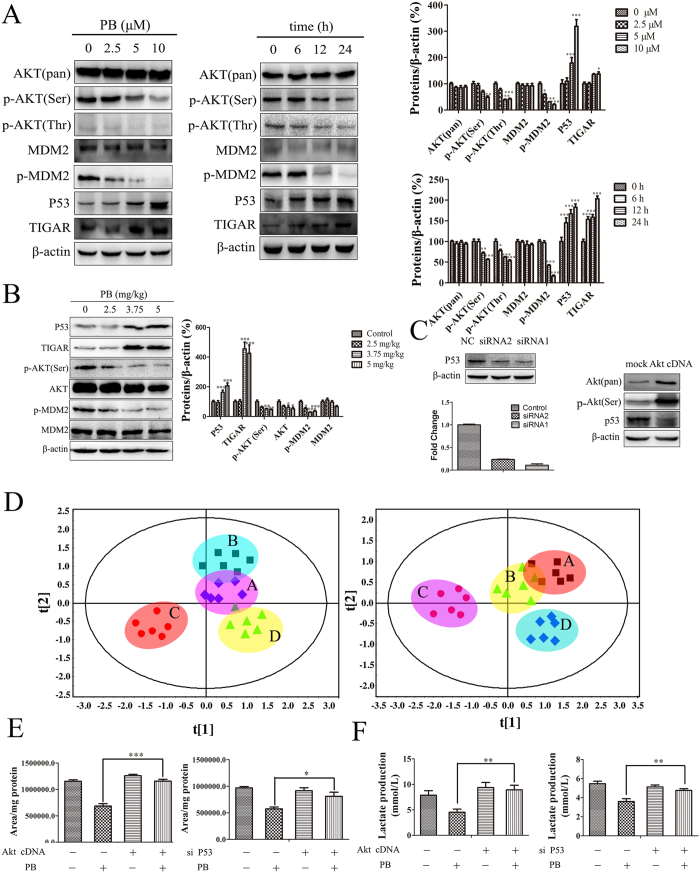
The effects of PB on metabolic pattern disruption were regulated through the Akt-P53 pathway. (**A**) The protein levels of AKT, p-AKT (Ser), p-AKT (Thr), MDM2, p-MDM2, p53 and TIGAR in HepG2 cells treated with various concentrations of PB and 5 μM PB for different time intervals, as determined by western blotting. (**B**) The protein levels of AKT, p-AKT (Ser), MDM2, p-MDM2, p53 and TIGAR in mice treated with various concentrations of PB, as determined by western blotting. (**C**) The gene and protein levels of p53 and the protein expression of AKT and p-AKT (Ser) after HepG2 cells were transfected with p53 siRNA or Akt cDNA. (**D**) The metabolic profile data of HepG2 cells after treatment with 5 μM PB for 24 h in the presence or absence of P53 siRNA or Akt cDNA, as measured by GC-MS. (Left: A-mock cDNA B-Akt cDNA C-mock cDNA + PB treatment D-Akt cDNA + PB treatment Right: A-NC siRNA B-P53 siRNA C-NC siRNA + PB treatment D-P53 siRNA + PB treatment) (**E**) The change in peak area for lactate in HepG2 cells after treatment with 5 μM PB for 24 h in the presence or absence of P53 siRNA or Akt cDNA. (**F**) The lactate level in the medium of HepG2 cells after treatment with 5 μM PB for 24 h in the presence or absence of P53 siRNA or Akt cDNA, as determined by lactate assay kit.

**Figure 7:**
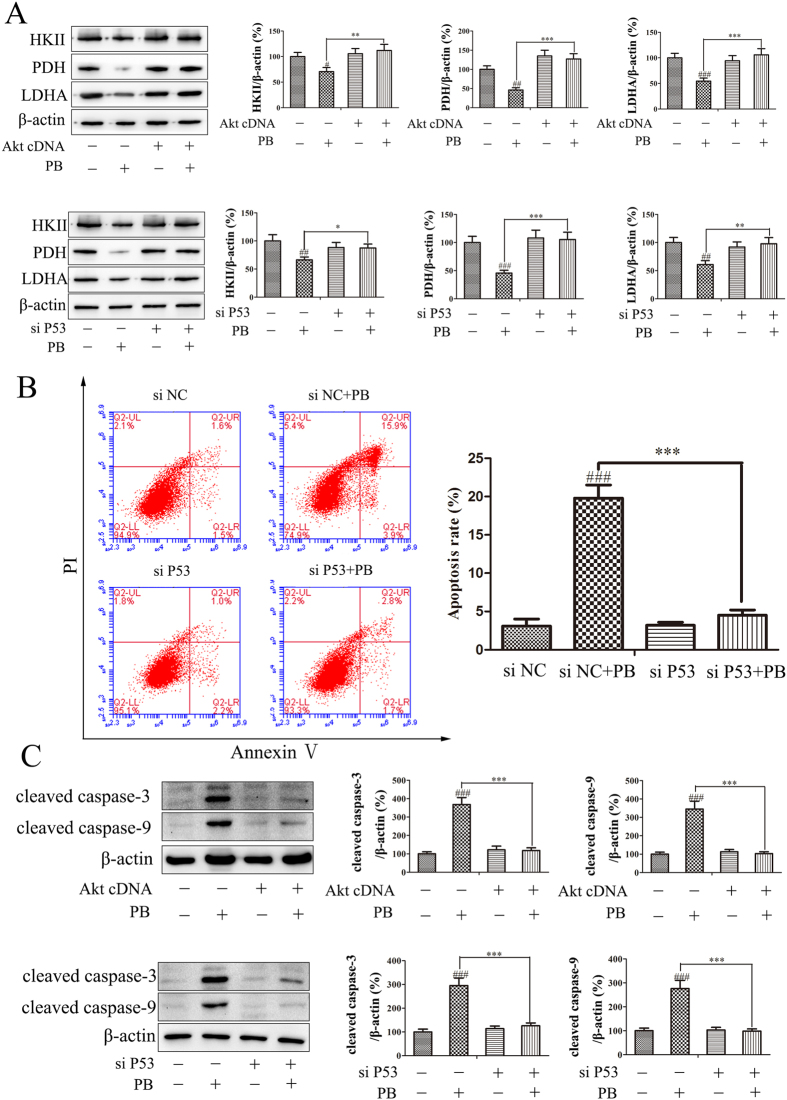
The effects of PB on metabolic pattern disruption were regulated through the Akt-P53 pathway. (**A**) HKII, PDH and LDHA levels in HepG2 cells after treatment with 5 μM PB for 24 h in the presence or absence of P53 siRNA or Akt cDNA, determined by western blotting. (**B**) The apoptotic rate of HepG2 cells transfected with NC siRNA or P53 siRNA together with treatment with 5 μM PB. (**C**) The protein levels of cleaved caspase-9 and cleaved caspase-3 in HepG2 cells after treatment with 5 μM PB for 24 h in the presence or absence of P53 siRNA or Akt cDNA.

As has been previously demonstrated, the Akt and p53 proteins are involved in changes in glycolytic activity and metabolism, as well as cell survival^18^. To further clarify the underlying mechanism of the changes in metabolic patterns characterized by activated apoptosis and decreased glycolysis, we investigated the role of the Akt-p53 pathway in these observed effects caused by PB. The protein expression of phospho-Akt (Ser 473), phospho-Akt (Thr308) and phospho-MDM2 all decreased in dose- and time- dependent manners in response to PB treatment, while the total expression of Akt and MDM2 showed little change. P53 and TIGAR were increased after the HepG2 cells were treated with PB (Fig. 6A). The decreased expression of phospho-Akt (Ser 473) and phospho-MDM2 and the increased expression of p53 and TIGAR were also detected in PB-treated mice (Fig. 6B). To confirm the role of Akt and p53 in these processes, additional experiments were conducted. When HepG2 cells were transfected with p53 siRNA or active Akt cDNA, the gene and protein levels of p53 both decreased (p53 siRNA treatment) and the protein level of Akt and phospho-Akt (Ser 473) increased along with a decreased level of p53 (active Akt cDNA treatment), providing proof that p53 siRNA and Akt cDNA were successfully transfected into cells and demonstrating the negative regulation of Akt on p53 (Fig. 6C). Subsequently, the metabolic traits of HepG2 cells transfected with Akt cDNA or p53 siRNA was measured. As shown in Fig. 6D, HepG2 cells transfected with Akt cDNA or p53 siRNA showed results closer to those of the control cells than to those of the cells transfected with mock cDNA or NC siRNA under the PB administration conditions, verifying the role of Akt and p53 in regulating the metabolic patterns observed here. The decreased lactate production triggered by PB was also reversed, as reflected by the quantitative data obtained from the peak areas (Fig. 6E), and the changes in lactic acid in the medium were consistent with those measured in the cells (Fig. 6F). In addition, the decreased expression of HKII, PDH and LDHA was rescued after Akt cDNA or p53 siRNA transfection, as shown by the western blotting analysis (Fig. 7A). Moreover, fewer apoptotic cells were observed in the p53 siRNA group than in the NC siRNA group when HepG2 cells were treated with 5 μM PB (Fig. 7B), and the increased expression of cleaved caspase-3 and cleaved caspase-9 was abrogated after Akt cDNA or p53 siRNA transfection (Fig. 7C). Therefore, these results suggest that Akt and p53 play crucial roles in the PB-mediated changes in metabolic patterns in tumour cells.

## Discussion

PB is a natural withanolide, and its analogues have been reported to exhibit cytotoxicity against many types of cancer cells through diverse mechanisms^19^,^20^. In our previous study, PB was found to inhibit the proliferation of cancer cells^16^. To confirm the anticancer actions of PB, HCC cells were chosen for the present study, and the results obtained verified that PB has strong cytotoxic effects on HepG2 cells. Furthermore, the antitumour effects of PB were confirmed in mice bearing H22 cells. The results demonstrate that PB obviously inhibit tumour growth and suggest that it has minimal side effects in mice, as reflected by the H&E staining results. Although the anticancer effects of PB *in vitro* and *in vivo* were verified, its underlying molecular mechanism was still unclear.

Metabolomics allows for a high-throughput analysis of cellular compounds with low molecular mass, which can reflect metabolic shifts in physiological processes and may reveal the underlying mechanisms related to processes induced by external factors^21^. To explore the mechanism by which PB induces cell death, a metabolomics analysis was used to evaluate the metabolic changes induced by PB. As shown by the results of the metabolomic analysis, PB severely disturbed metabolic patterns *in vitro* and *in vivo*, and the heat map also indicates that PB exposure dramatically changed the metabolite levels in HepG2 cells (Fig. S4). The pathway analysis indicate glycolysis plays a significant role in the perturbed pathway induced by PB. Statistical processing and chemometric procedures suggest that potential biomarkers (e.g., lactic acid and glucose) could be used to differentiate the control and the PB treated samples. Notably, lactate was the most important variable compound reflected by the OPLS-DA plot, indicating that glycolysis plays an important role in the PB-induced pathway changes. Subsequently, molecular biological methods were used to verify these phenomena, and decreases in genes and proteins related to glycolysis were detected in HepG2 cells and mice via qRT-PCR and western blotting. Taken together, these results show that metabolomics can provide powerful clues in the process of looking for targeted pathways and that molecular biological methods can be used to confirm the predicted mechanisms.

Metabolomics provides a valuable platform for the investigation of the metabolic perturbations in HCC cells, and glycolysis has recently been added to the list of hallmarks that define cancer. As cellular metabolism is a complex network of interdependent pathways, local alterations will have an impact on overall tumour metabolism. Therefore, the glycolytic pathway has been highlighted in relation to its role in increased rates of cell proliferation^22^. In addition, glycolysis is a universal property of malignant cells, and it induces acidification of the tumour environment, favouring the development of a more aggressive and invasive phenotype^5^. Therefore, downregulating the level of glycolysis can trigger antitumour effects^23^,^24^,^25^, and the control of major glycolytic enzymes and metabolic products is closely related to the fate of cancer cells^26^,^27^,^28^. For example, the inhibitor of glycolysis 2-DG has been successfully applied to induce apoptosis^29^. In our experiment, an elevated expression of cleaved caspase-3/7/9 and PARP was observed (Fig. S5), demonstrating that decreasing the level of glycolysis could induce apoptosis. From the metabolomic data, PB was shown to disturb the metabolic pattern of HepG2 cells and mice and to decrease the level of glycolysis. Additionally, a decrease in proteins and genes related to glycolysis was measured using molecular biological method. Therefore, whether apoptosis could be induced by PB was an intriguing question for us. Our results show that PB promoted the cleavage of caspase-3, -7, -9 and PARP in HepG2 cells and mice. Previous findings have demonstrated that mitochondrial damage can trigger mitochondrial membrane permeabilization (MMP) followed by the release of apoptogenic factors, including cytochrome *c*, leading to caspase activation and thus promoting apoptosis^30^. The release of cytochrome *c* is tightly regulated by the Bcl-2 family of proteins that control MMP^31^. Therefore, mitochondrial apoptosis is closely correlated to the life and death of cancer cells^32^. In our experiment, a decreased mitochondrial membrane potential (ΔΨm) and an increased Bax/Bcl-2 protein expression ratio were also observed, demonstrating that mitochondrial dysfunction is involved in the PB-induced apoptotic response^33^. P53 is a common tumour suppressor gene and can induce apoptosis and cell cycle arrest in many types of cancer cells^34^,^35^. In response to apoptotic stimuli, a fraction of the p53 pool rapidly translocates to the mitochondria and binds to anti-apoptotic Bcl-2 family proteins, releasing the pro-apoptotic effectors Bak/Bax from their complex with the anti-apoptotic proteins^36^. Subsequently, the released Bak and Bax induce lipid pore formation in the outer mitochondrial membrane, which elicits cytochrome *c* release and triggers apoptosis^37^,^38^,^39^. In addition to mediating apoptosis, p53 can also modulate glycolysis via cytochrome *c* oxidase 2 (SCO2) and TP53-induced glycolysis and apoptosis regulator (TIGAR)^6^,^7^. Moreover, a large fraction of human cancers is dependent on aberrant survival signalling pathways, such as the PI3K/Akt pathway, which are highly associated with energy metabolism and a classic biochemical phenotype. Additionally, PI3K/Akt pathway-mediated HKII expression up-regulates the Warburg effect and further facilitates tumour growth^17^. There have been other reports showing that Akt stimulates aerobic glycolysis in cancer cells and that the activity of Akt renders cancer cells dependent on aerobic glycolysis for continued growth and survival^40^,^41^,^42^. Additionally, the Akt-mediated phosphorylation of MDM2 also promotes the nuclear localization of MDM2 and inhibits interactions between MDM2 and p53 as well as the ubiquitination of p53, thereby decreasing p53 stability^43^,^44^. In our research, the nuclear localization of MDM2 when HepG2 cells were treated with PB showed no significant difference compared to the control (Fig. S6), but the expression of p-MDM2 decreased obviously, which suggested the p53 stability was mainly mediated by phosphorylation of MDM2 at Ser^186^. These findings show that the Akt-p53 pathway is important in the physiological processes of apoptosis and glycolysis. In our study, increased levels of p53 and decreased levels of p-Akt were found in response to PB treatment. When HepG2 cells were transfected with Akt cDNA or p53 siRNA, the attenuation of glycolysis and enhancement of apoptosis were reversed. The metabolomic data from cells transfected with Akt cDNA or p53 siRNA were also measured. The compounds related to glycolysis were selected and a PCA plot was made. The HepG2 cells transfected with Akt cDNA or p53 siRNA clustered closer the control cells on the plot than to the cells transfected with mock cDNA or NC siRNA after incubation with PB. This result verifies that Akt and p53 are involved in the perturbation of metabolic patterns induced by PB. In summary, the roles for p53 and Akt were confirmed in the reduced glycolysis and enhanced apoptosis triggered by PB using metabolomic and molecular biological methods together.

**Figure 8:**
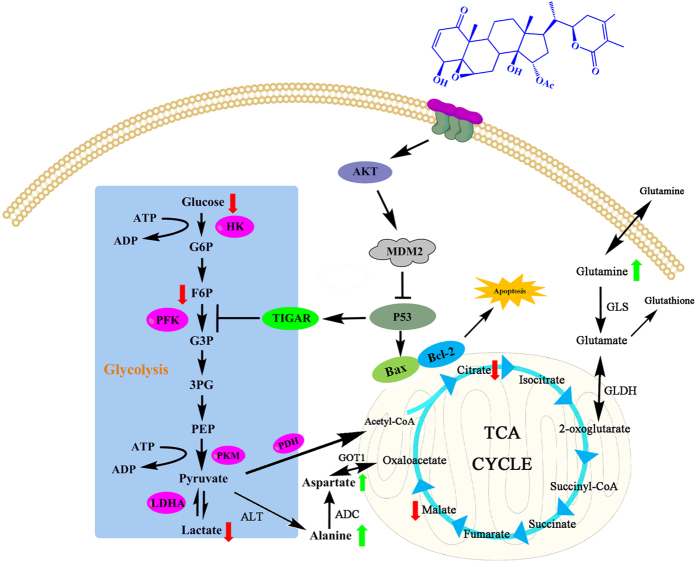
The signalling pathway changes triggered by PB. The red arrow represents the decreased metabolites and the green arrow represents the increased metabolites, as measured by GC/MS.

In this study, the molecular mechanism underlying the anticancer effects of PB on HepG2 cells was investigated using a combination of metabolomic and molecular biological methods. The signalling pathway shown in Fig. 8 illustrates how PB affects the metabolic pattern and exhibits antitumour effects. In conclusion, this study demonstrates that PB decreases glycolysis and induces apoptosis in HCC cells. The underlying molecular mechanism of these effects was effectively and quickly predicted based on the metabolomics analysis and was confirmed by means of molecular biological methods. Moreover, the Akt-p53 pathway was shown to regulate the effect of PB on metabolic pattern disturbances characterized by decreased glycolysis and activated apoptosis. All of these results indicate that metabolic profiling methods have distinct advantages in the exploration of targeted pathways and biomarkers and that PB could be a promising alternative option for cancer therapy.

## Materials and Methods

### Cell type

Cancer cells from the human hepatocellular carcinoma HepG2 cell line and cells from the human normal liver LO2 cell line were purchased from the Cell Bank of Shanghai Institute of Biochemistry and Cell Biology, Chinese Academy of Sciences (Shanghai, China). Mouse H22 hepatoma cells were purchased from the American Type Culture Collection (ATCC).

### Reagents

PB was isolated from *Physalis angulata* var. *villosa* in our laboratory. Samples containing 95% or higher concentrations of PB were used in all of our experiments. PB was dissolved in dimethyl sulfoxide (DMSO) to obtain a stock solution of 20 mmol/L and stored at room temperature. Before each experiment, the stock solution of PB was diluted with DMEM (HyClone SH30243.01B) to different working concentrations. Control groups were treated with the same amount of DMSO (0.1%) in the corresponding experiments. Cleaved caspase-3 rabbit mAb, caspase-3 antibody, caspase-9 antibody, cleaved caspase-9 antibody, caspase-7 antibody, cleaved caspase-7 antibody, PARP antibody, cleaved-PARP antibody, hexokinase I (C35C4) rabbit mAb, hexokinase II rabbit mAb, LDHA (C4B5) rabbit mAb, PFKP (D4B2) rabbit mAb, PKM1/2 (C103A3) rabbit mAb, PKM2 (D78A4) XP rabbit mAb, pyruvate dehydrogenase (C54G1) rabbit mAb, phospho-MDM2 (Ser166) antibody, p53 (7F5) rabbit mAb, Akt (pan) (C67E7) rabbit mAb, phospho-Akt (Ser473) (D9E) XP rabbit mAb, phospho-Akt (Thr308) (D25E6) XP rabbit mAb, Bax (D2E11) rabbit mAb and Bcl-2 (D55G8) rabbit mAb antibodies were purchased from Cell Signaling Technology (Danvers, MA, USA). TIGAR (H-120): sc-67274 and MDM2 (SMP14): sc-965 antibodies were purchased from Santa Cruz Biotechnology, Inc. Anti-cleaved PARP and anti-LDHA antibodies were purchased from Abcam, and β-actin was purchased from Vazyme (Nan Jing, China).

### Cell viability and tumour colony forming assays

The cell viability of HepG2 and LO2 cells was measured via MTT assay. Cells were seeded in 96-well culture plates (5 × 10^3^ cells/well). After incubation overnight, the HepG2 cells were treated with different concentrations of PB for 24, 48 or 72 h and LO2 cells were treated with various concentrations of PB for 24 h. MTT (5 mg/ml) was dissolved in PBS and filter-sterilized. Then, 20 μl of the prepared MTT solution was added to each well and the cells were incubated for another four hours until a purple precipitate was visible. The formed formazan crystals were dissolved in DMSO (150 μl/well) by continuous shaking for 10 min. The absorbance was measured on an ELISA reader (Spectra Max Plus384, Molecular Devices, Sunnyvale, CA) at a test wavelength of 570 nm and a reference wavelength of 650 nm. Cell viability was calculated using the following formula:


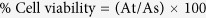


At and As represent the absorbance of the test substances and solvent control, respectively.

For colony-forming assays, cells were plated in 6-well culture plates (1 × 10^3^ cells per well) and treated with different concentrations of PB or 0.1% DMSO for 10 days. The cells in the plates were then stained with a crystal violet solution (Sigma-Aldrich, St. Louis, MO), and the photographs of the colonies were taken manually.

### Annexin V-FITC/PI staining assay and Hoechst 33258 staining for cell apoptosis

The HepG2 cells that underwent apoptosis were evaluated via flow cytometry and fluorescence microscopy. HepG2 cells were treated with various concentrations of PB or 0.1% DMSO for 24 h. For flow cytometry, 1 × 10^6^ HepG2 cells in 500 μl of binding buffer were stained with 5 μl of Annexin V-FITC and 5 μl of propidium iodide at room temperature in the dark for 15 min. Cells were then analysed via flow cytometry (488 nm excitation and 600 nm emission filters) using a BD FACS Calibur flow cytometer (Becton & Dickinson Company, Franklin Lakes, NJ). For Hoechst 33258 staining assay, cells were stained with Hoechst 33258 for 10 min. The cells were then washed with PBS twice and observed under a fluorescence microscope (LEICA DMI3000 B, Germany).

### 5-ethynyl-20-deoxyuridine (EdU) incorporation assay

Following the instructions provided with the EdU labelling/detection kit (RiboBio, Guangzhou, China), HepG2 cells were incubated in a final volume of 2 mL of complete medium at 2.5 × 10^5^ cells per well on a glass-bottom dish. Following incubation for 18 h, the medium above the cells was discarded and replaced with fresh medium containing PB at different concentrations (2.5 μM, 5 μM and 10 μM) or DMSO (<0.1%) as the vehicle. Twenty hours later, 100 μL of fresh medium containing of 50 μM EdU labelling agent was added to the cell cultures, which were then incubated for another 8 h at 37 °C under a 5% CO_2_ atmosphere. Then, cells were incubated with glycine for 5 min after being fixed with 4% paraformaldehyde (pH 7.4) for 30 min. After being washed with PBS, the cells were stained with the anti-EdU working solution at room temperature for 30 min. Following a wash with 0.5% Triton X-100 in PBS, the cells were incubated with 5 μg/ml of Hoechst 33342 at room temperature for 30 min, and then, HepG2 cells were followed by observing them under a confocal laser scanning microscope (TCS SP2, Leica Microsystems, Germany; LSM710, Carl Zeiss, Germany).

### Western blotting analysis

HepG2 cells were incubated with different concentrations of PB or 0.1% DMSO for 24 h. After being harvested with trypsin, cells were treated with a 1 × RIPA lysis buffer (50 mM Tris–HCl (pH 7.4), 150 mM NaCl, 0.25% deoxycholic acid, 1% NP-40, 1 mM EDTA and the protease inhibitor PMSF) (Amresco, Solon, USA) to extract the total proteins. Then, aliquots of the proteins from the total cell lysates (4 to 40 μg/lane) were separated via sodium dodecyl sulfate (8%, 10% or 12%) polyacrylamide gel electrophoresis (SDS-PAGE, Bio-Rad Laboratories, Hercules, CA), wet-transferred to a PVDF membrane (Bio-Rad Laboratories, Hercules, CA) and blotted with primary antibodies specific for cleaved caspase-3, caspase-3, PARP, cleaved PARP, caspase-9, cleaved caspase-9, caspase-7, cleaved caspase-7, HKI, HKII, PFKP, PDH, LDHA, PKM1/2, PKM2, Akt, p-Akt (Thr308), p-Akt (Ser473) MDM2, p-MDM2, p53, TIGAR, Bax, Bcl-2 and β-actin, which was used as the internal standard, overnight. Samples were then probed with secondary isotype-specific antibodies for an additional hour at 37 °C. Bound immuno-complexes were detected using the ChemiDOC™ XRS+system (Bio-Rad Laboratories, Hercules, CA).

### Metabolomics analysis

To help the extraction of intracellular metabolites, the HepG2 and LO2 cell samples (n = 6) were first subjected to three freeze–melt cycles (freezing at −80 °C for 60 min; melting at 37 °C for 20 min). Next, 900 μl of methanol containing 1.5 μg of (2C^13^)-myristic acid as an internal standard (IS) was added to the cells. For the animal samples (n = 7), 20–30 mg of tissue was added to 800 μl of 80% methanol, and 100 μl of plasma (n = 6) was added to 400 μl of methanol containing an IS. All samples were vortexed vigorously for 5 min and then centrifuged at 12,000 × *g* for 10 min. Next, the supernatant (350 μl) was evaporated to dryness using an SPD2010–230 SpeedVac Concentrator (Thermo Savant, Holbrook, USA). When the sample was completely dried, 30 μl of methoxyamine in pyridine (10 mg/mL) was added to the dried residue and vigorously vortex-mixed for 3 min. The methoximation reaction continued for at least 16 h at room temperature, followed by trimethylsilylation for 1 h by adding 30 μl of N-methyl-N-(trimethylsilyl)trifluoroacetamide (MSTFA) with 1% TMCS as the catalyst. Finally, the solution was vortex-mixed again for 5 min after the external standard (methyl myristate in heptane (30 μg/mL)) was added into each GC vial. Then, GC/MS metabolomic analyses were performed. Briefly, the derivatized sample (0.5 μl) was injected into a 10 m × 0.18 mm ID fused-silica capillary column chemically bonded with a 0.18 μm DB-5 MS stationary phase (J&W Scientific) in an Agilent 6890 GC system, and the analytes in the eluent were detected using MS (Leco Corp., St. Joseph, MI, USA). The mass spectrum was scanned and collected (50–680 *m*/*z*) at a rate of 30 spectra/s after a 170 s solvent delay^21^. Automatic peak detection and mass spectral deconvolution were performed using software (Leco, version 3.25) as previously reported^45^.

The metabolites in different types of samples were identified separately by comparing the mass spectra and retention times of the detected compounds with those of reference standards available in the National Institute of Standards and Technology (NIST) library 2.0 (2008), Wiley 9 database (Wiley–VCH Verlag GmbH & Co. KGaA, Weinheim, Germany), and an in-house mass spectra library database established by the Umeå Plant Science Center, Swedish University of Agricultural Sciences (Umeå, Sweden).

### Quantitative real-time RT-PCR

Levels of mRNA expression were analysed using RT-PCR assay. Total RNA was isolated from HepG2 cells using an EASYspin Plus tissue/cell RNA extraction kit (Aidlab Biotechnologies Co. Ltd). RNA was quantified by measuring absorption at 260 nm, and 1 μg RNA was reverse transcribed to cDNA by using the Transcriptor First Strand cDNA Synthesis Kit (Roche Diagnostics, Basel, Switzerland)^46^. Thermal cycling conditions included an initial denaturation at 95 °C for 5 min, followed by 40 cycles of denaturation (10 s at 95 °C), annealing (15 s at 60 °C) and extension (15 s at 72 °C with a single fluorescence measurement), a melting curve programme (60–95 °C with a 0.11 °C/s heat increase and continuous fluorescence measurement) and a cooling step to 40 °C. The Δ cycle threshold method was used for the calculation of the relative differences in mRNA abundance with a LightCycler 480 system (Roche Molecular Biochemicals, Mannheim, Germany). The data were normalized to the expression of β-actin. The results are expressed as fold changes. The RT-PCR primers used in this study are listed in Table S1.

### Measurement of glucose uptake

Glucose in the medium was quantitated via a glucose assay kit (Jian Cheng, Nan Jing) using a standard curve prepared with serial dilutions of low-glucose DMEM (5.55 mmol/L glucose) into glucose-free DMEM. The absorbance was measured on an ELISA reader (SpectraMax Plus384, Molecular Devices) at a test wavelength of 505 nm. The concentration of glucose uptake in each sample was calculated using the following formula:


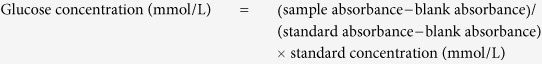


### Measurement of lactate production

The lactate level was measured via a lactic acid assay kit (Jian Cheng, Nan Jing) using a standard curve made with serial dilutions of 3 mmol/L-lactate. The absorbance was measured on an ELISA reader (SpectraMax Plus 384, Molecular Devices) at a test wavelength of 530 nm. The concentration of lactate in each sample was calculated using the following formula:


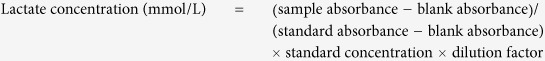


### Plasmid and siRNA transient transfection

The pcDNA3 T7 Akt1 cDNA was purchased from Addgene (Beijing Zhongyuan Ltd China). SiRNA targeting p53 was purchased from Biomics (Biomics Biotechnologies, Nantong, China). For siRNA transfection, cells were seeded in 6-well plates. P53 siRNA (100 nmol/L) was introduced into the cells using Lipofectamine^®^ 3000 Transfection Reagent (Invitrogen) according to the manufacturer’s recommendations. Then, the cells were exposed to DMEM with or without PB and harvested for further experiments.

For plasmid transfection, plasmid DNA (2 μg) was introduced into the HepG2 cells using Lipofectamine^®^ 3000 Transfection Reagent (Invitrogen) according to the manufacturer’s recommendations. Cells were then exposed to PB or the vehicle (0.1% DMSO) in 2 mL DMEM and harvested for further experiments.

### Immunofluorescence

HepG2 cells in 6-cm dish were treated with or without PB for 24 h. The cells were fixed with 4% paraformaldehyde for 15 min and blocked with 5% BSA for 1 h. Incubation with primary antibodies against MDM2 was done overnight at 4 °C. The cells were then incubated with Alexa Flour 488-conjugated secondary antibody for 2 h at room temperature in the dark. The nuclei were stained with DAPI (Beyotime, Haimen, China) 10 min before imaging. A laser scanning confocal microscope LSM 700 (Carl Zeiss, Oberkochen, Germany) was used for co-localization analysis.

### *In vivo* studies

H22 cell inoculation was performed in 5-week-old Institute of Cancer Research (ICR) male mice. Twenty-four hours later, the mice were divided randomly into five groups (n = 7) and intraperitoneally administered daily doses of saline (control group); PB at 2.5, 3.75 or 5 mg/kg; or adriamycin (Adr) 2 mg/kg. Body weight was measured every day. On the 14th day, the mice were killed, and the tumour was removed and weighed, with one part being stored at −80 °C and the others fixed in 4% paraformaldehyde (pH 7.4). The tumour inhibition ratio was calculated based on tumour weight. For histological examinations, tissues embedded in paraffin were stained with haematoxylin and eosin (H&E). Apoptosis was evaluated via TUNEL assay, and the Ki-67 assay was performed to assess cancer cell proliferation. During the experiments, mice were cared for and handled in strict accordance with the guidelines of the Animal Ethics Committee of China Pharmaceutical University and the National Institutes of Health (NIH) standard guidelines for the Care and Use of Laboratory Animals. And all experimental protocols were approved by the Animal Ethics Committee of China Pharmaceutical University.

### Multivariate data analysis

All the relative quantitative peak areas of each detected peak in cells and animals samples were normalized to myristic-1, 2-^13^C2 acid, the stable isotope IS (RSD < 5%), before a multivariate statistical analysis by using SIMCA-P 12.0 software (Umetrics, Umeå, Sweden). Heatmaps were produced by using R-Project that is available online at: http://www.r-project.org/ (Vanderbilt University, Nashville Tennessee, USA). In the process of metabolic data analysis, a principal component analysis (PCA) and a orthogonal projection to latent structures discriminant analysis (OPLS-DA) were employed to maximize covariance between the acquired GC/MS data and the response variable. Samples from the same groups were grouped together for PCA and OPLS–DA modeling. The PCA and OPLS–DA results were shown as scores plots to visualize sample clustering and to indicate sample similarity and difference between each other. The model goodness of fit was evaluated using three quantitative parameters; i.e., R^2^X is the explained variation in X, R^2^Y is the explained variation in Y, and Q^2^Y is the predicted variation in Y. Cross-validation was used throughout to determine the principal component number^47^,^48^, which was determined once the Q^2^Y value decreased continuously. Permutation tests were performed with 100 iterations to validate the model. In addition, S-plot was constructed to reveal variables that contributed to the group separation. Lactate and some other metabolites between the treated and control groups were screened in the column and then validated using a one-way analysis of variance with a significance level of 0.05. The impact of physapubenolide on metabolic pathways was evaluated based on a tool for metabolomic data analysis, which is available online (http://www.metaboanalyst.ca/MetaboAnalyst/)^49^. The Pathway Analysis help researchers identify the most relevant pathways involved in the conditions under research.

### Statistical analysis

All experiments were conducted more than three times. The results were analysed by using GraphPad Prism version 5.0 to perform one-way or two-way ANOVAs (GraphPad Software, San Diego, CA, USA). The results are given as the mean ± SD. A p value less than 0.05 was considered statistically significant.

## Additional Information

**How to cite this article**: Ma, T. *et al*. Metabonomics applied in exploring the antitumour mechanism of physapubenolide on hepatocellular carcinoma cells by targeting glycolysis through the Akt-p53 pathway. *Sci. Rep.* **6**, 29926; doi: 10.1038/srep29926 (2016).

## Supplementary Material

Supplementary Information
