# Supplementary material for: Metabonomics applied in exploring the antitumour mechanism of physapubenolide on hepatocellular carcinoma cells by targeting glycolysis through the Akt-p53 pathway
Source: Sci Rep. 2016 Jul 15;6:29926. doi: 10.1038/srep29926 (PMC4945937; doi:10.1038/srep29926)

**Metabonomics applied in exploring the antitumour mechanism of physapubenolide on hepatocellular carcinoma cells by targeting glycolysis through the Akt-p53 pathway**

Ting Ma, Bo-Yi Fan, Chao Zhang, Hui-Jun Zhao, Chao Han，Cai-Yun Gao, Jian-Guang Luo* & Ling-Yi Kong*

State Key Laboratory of Natural Medicines, Department of Natural Medicinal Chemistry, China Pharmaceutical University, 24 Tong Jia Xiang, Nanjing 210009, China

Corresponding authors

* Ling-Yi Kong:

E-mail: cpu_lykong@126.com.

Tel/Fax: +86 25 8327 1405.

* Jian-guang Luo:

E-mail: luojg@cpu.edu.cn.

Tel/Fax: +86 25 8327 1402.

**Table S1**

Primers and annealing temperatures used for q RT-PCR

| Gene | Forward primer/ Reverse primer  (5′-3′) | Annealing temperature (°C) |
| --- | --- | --- |
| HKI | CATTCGTAAGGTCCATTC  / CTCCATGTGAACATTCTG | 60 |
| HKII | ACAATGGATGCCTAGATG  / AGGTACATTCCACTGATC | 60 |
| PKM2 | CCACTTGCAATTATTTGAGGA A  / GTGAGCAGACCTGCCAGACT | 60 |
| PFKP | ACCACCGATGATTCCATT  / CTTGAGCCACCACTGTTC | 60 |
| LDHA | TGGTTGAGAGTGCTTATG  / GCCTAAGATTCTTCATTATACT | 60 |
| PKM1/2 | CTATCCTCTGGAGGCTGTGC  / CCATGAGGTCTGTGGAGTGA | 60 |
| β-actin | GCGTGACATTAAGGAGAAG  / GAAGGAAGGCTGGAAGAG | 60 |
|  |  |  |

**Figure Captions**

**Figure S1.** The various tissues of treated and control mice after H&E staining.

Bar, 20 μm.

**Figure S2.** GC/MS chromatograms of plasma and tumour samples obtained from mice, and the changes in lactate levels revealed by differences in peak area.

**Figure S3.** (A) The PCA plot based on the intracellular metabolites in PB-treated or untreated HepG2 and LO2 cells and in the plasma of mice. a: A-LO2 control, B-HepG2 control, C-HepG2 PB-treated; b: red-LO2 control, green-LO2 PB-treated; c and d: changes in metabolic patterns detected in the plasma of mice. A-normal; B-control; C-Adr treated; D-PB treated; (B) The other intracellular metabolites changes in HepG2 cells determined by GC-MS.

**Figure S4.** The heat map of LO2 and HepG2 cells treated with or without 5 μM PB.

**Figure S5.** The protein expressions of cleaved PARP, cleaved caspase-3, cleaved caspase-7 and cleaved caspase-9 in HepG2 cells after treatment with 0, 10, 20 and 30 mM 2-DG.

**Figure S6.** Nuclear localization of MDM2 assay by immunofluorescence.


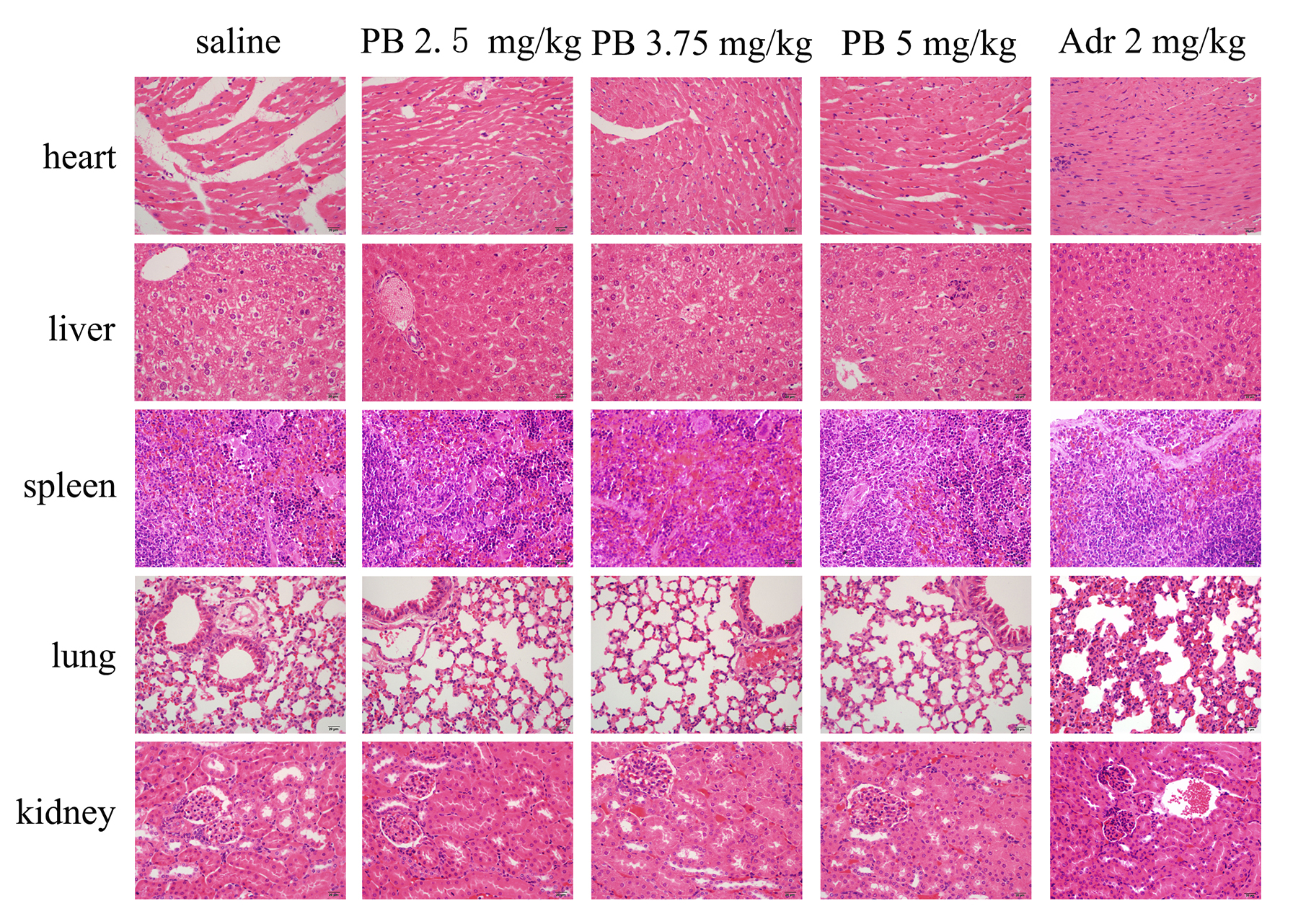


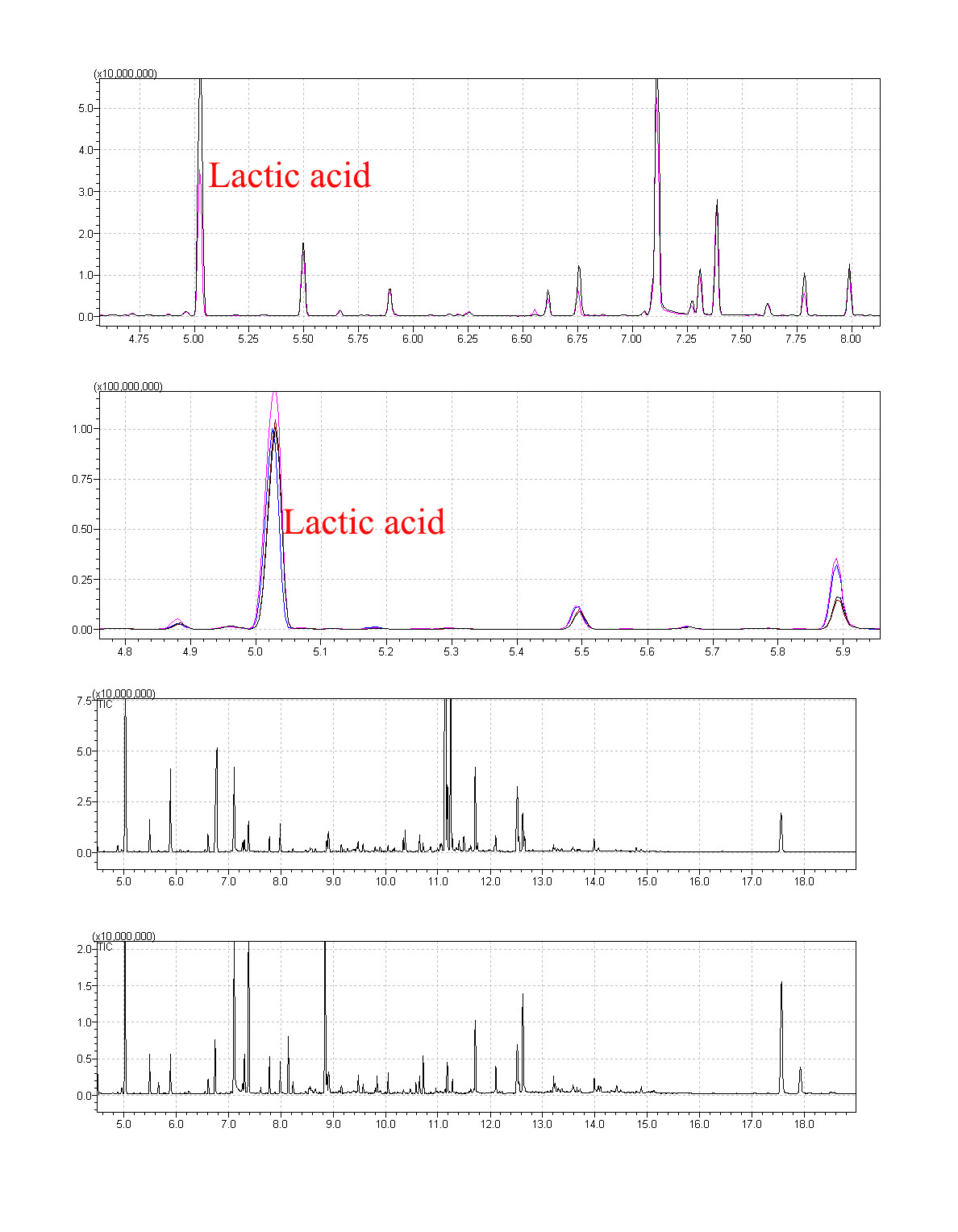


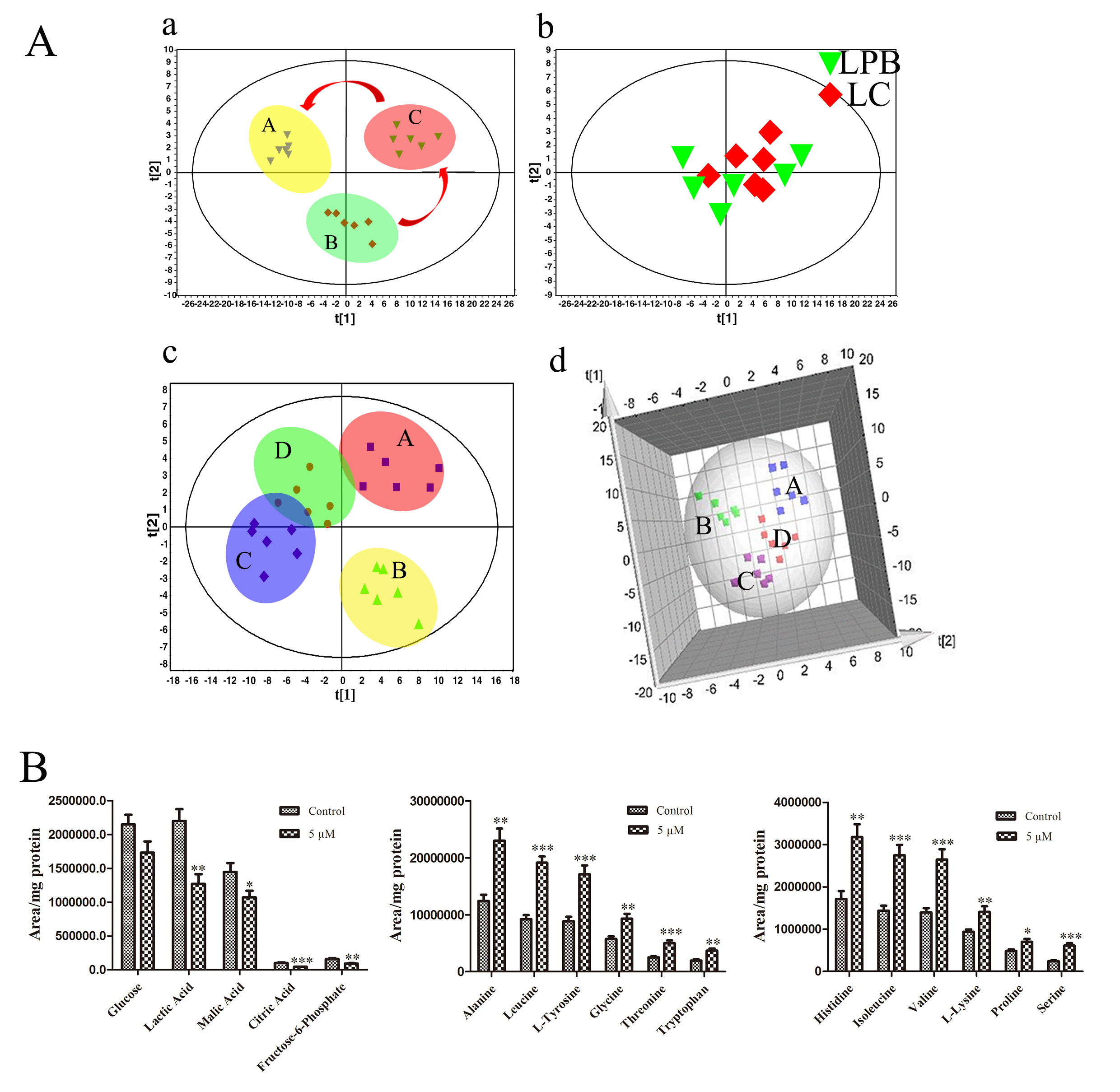


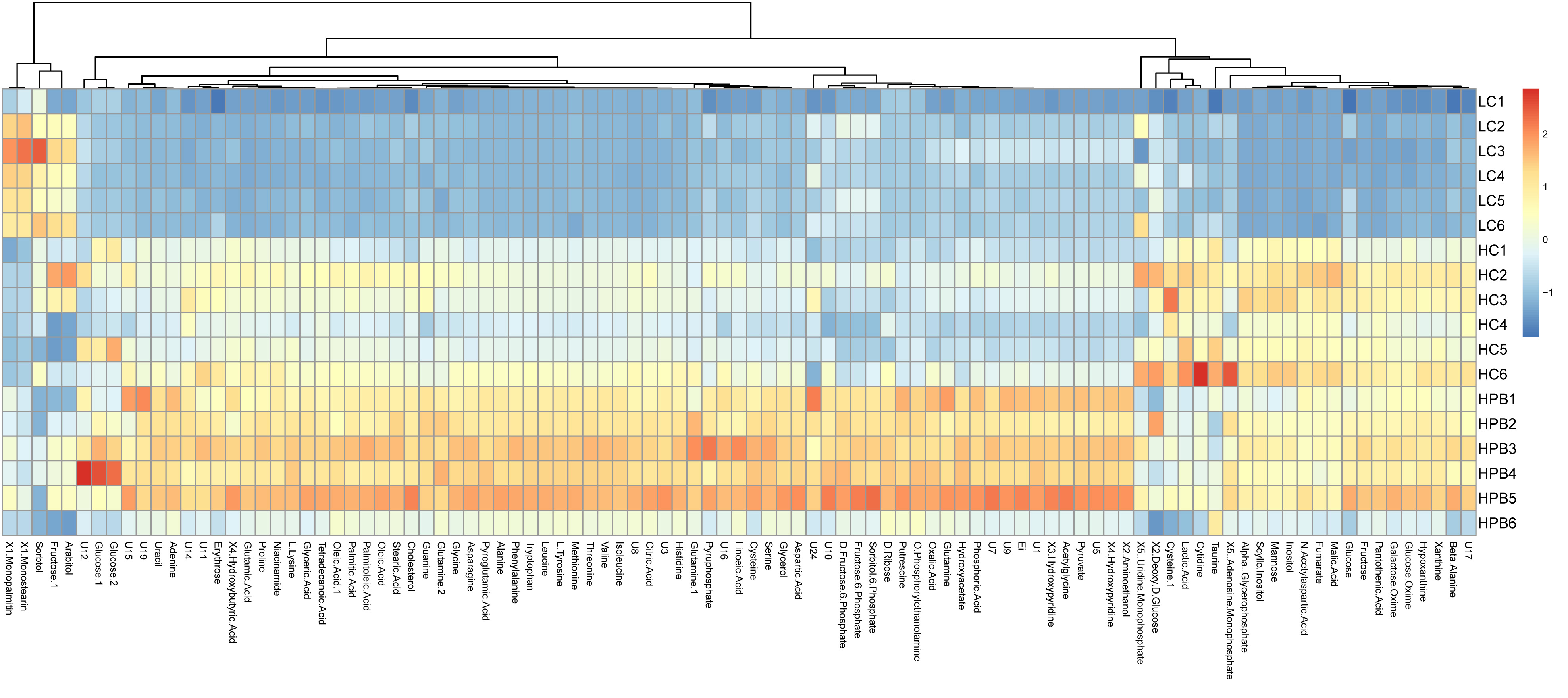


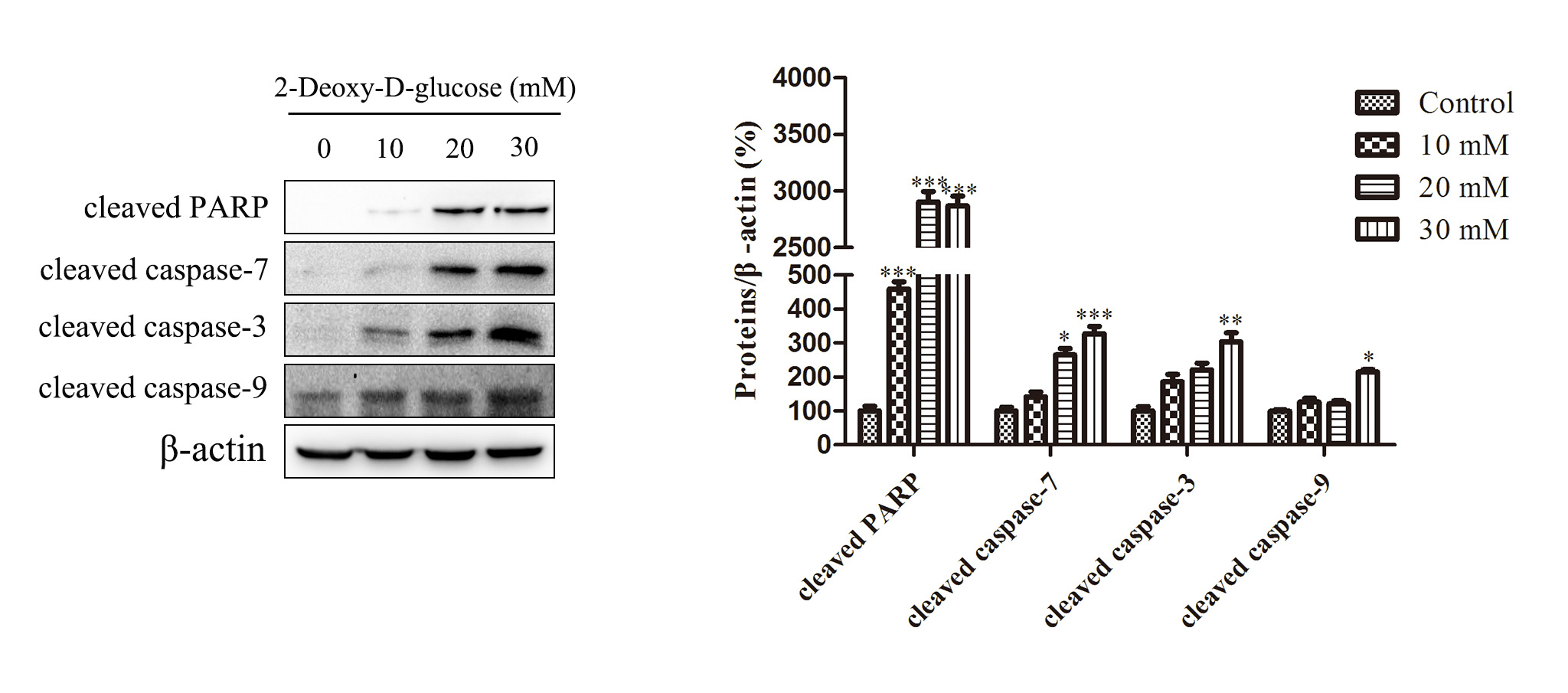


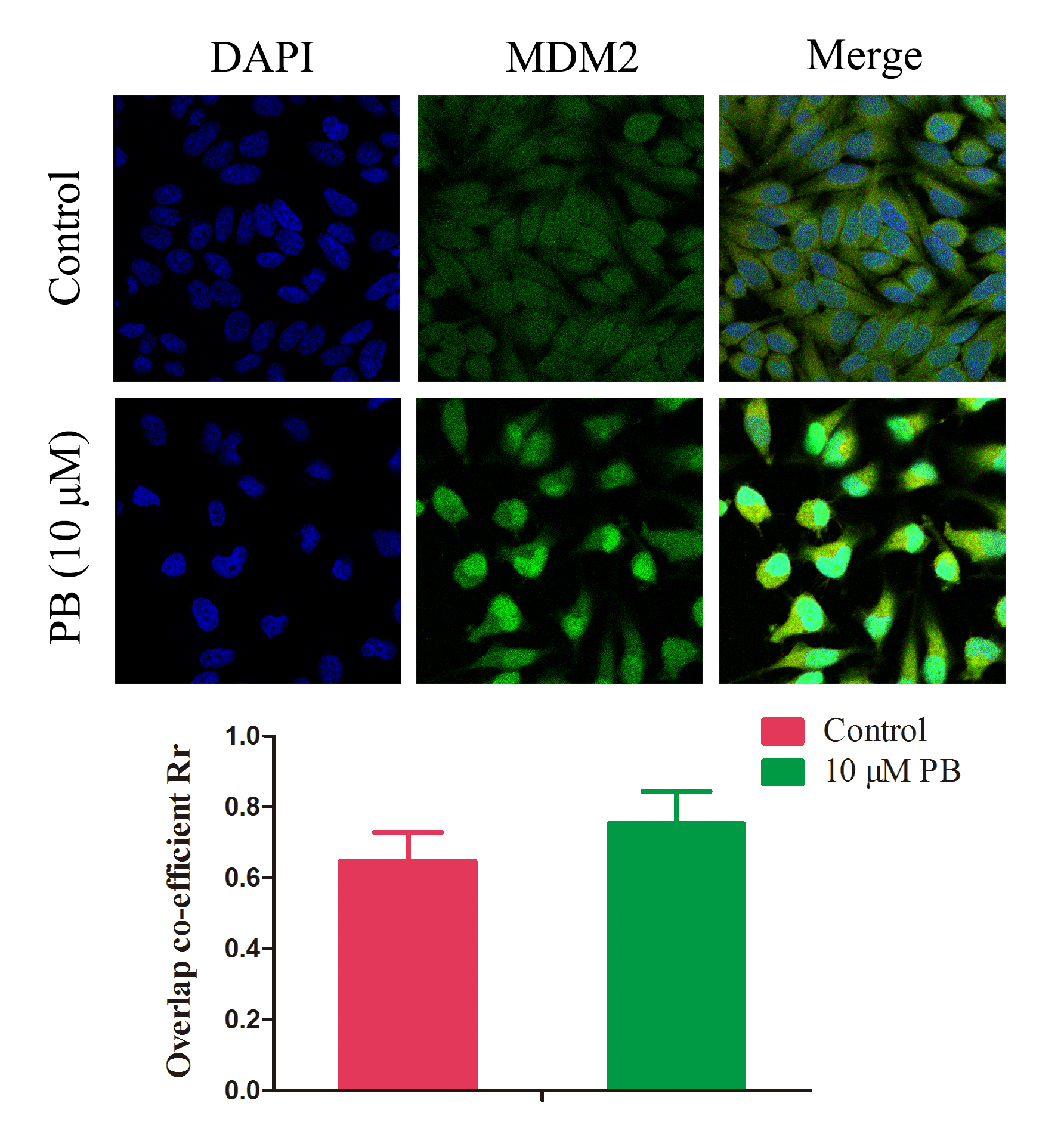

Supplement: Supplementary Information [file srep29926-s1.doc]
